# Case report of isolated aortic valve AL-amyloidosis following aortic valve replacement

**DOI:** 10.1093/ehjcr/ytaf393

**Published:** 2025-08-08

**Authors:** Rajin Choudhury, Victor H Jimenez-Zepeda, Etienne Mahe, Nowell M Fine

**Affiliations:** Division of Cardiology, Department of Cardiac Sciences, Libin Cardiovascular Institute of Alberta, University of Calgary, Calgary, Alberta, Canada T2N 1N4; Department of Medicine, Cumming School of Medicine, University of Calgary, Calgary, Alberta, Canada T2N 1N4; Department of Medicine, Cumming School of Medicine, University of Calgary, Calgary, Alberta, Canada T2N 1N4; Division of Medical Oncology and Hematology, Arthur J.E. Child Comprehensive Cancer Centre, University of Calgary, Calgary, Canada AB T2N 1N4; Department of Pathology and Laboratory Medicine, Department of Medicine, University of Calgary, Calgary, Canada AB T2N 1N4; Division of Cardiology, Department of Cardiac Sciences, Libin Cardiovascular Institute of Alberta, University of Calgary, Calgary, Alberta, Canada T2N 1N4; Department of Medicine, Cumming School of Medicine, University of Calgary, Calgary, Alberta, Canada T2N 1N4

**Keywords:** Cardiac AL-amyloidosis, Aortic stenosis, Aortic valve replacement, Case report

## Abstract

**Background:**

Light chain (AL)-amyloidosis is a haematologic malignancy where cardiac involvement confers a worse prognosis. There is a recognized association between aortic stenosis (AS) and transthyretin amyloidosis (ATTR) cardiomyopathy. However, there is no such reported association with AL amyloidosis.

**Case summary:**

We present a case of degenerative AS where the pathology analysis post-surgical replacement demonstrated amyloid deposits subsequently identified as AL subtype by mass spectrometry. Subsequent investigations demonstrated no myocardial or systemic involvement. Following multidisciplinary discussion, cardiac biopsy and chemotherapy were deferred given the isolated nature of aortic valve involvement and clinical stability.

**Discussion:**

Incidental detection of aortic valve AL-amyloidosis in this case demonstrates that such deposits cannot be assumed to be ATTR, and further work-up and amyloid typing is necessary.

Learning points

- Aortic stenosis is often associated with the transthyretin (ATTR) type of cardiac amyloidosis, although identifying aortic stenosis patients with cardiac amyloidosis can be challenging.
- Other types of amyloidosis, including the malignant light-chain (AL) amyloidosis can also occur with aortic stenosis, even in isolation of myocardial involvement.
- Multidisciplinary team approach to amyloidosis care is critical, especially for complex or unusual case presentations.

## Introduction

The coexistence of cardiac amyloidosis (CA) and aortic stenosis (AS) is well recognized, although diagnosing cardiac amyloidosis in the presence of AS can be challenging due to overlapping clinical features. Amyloid light chain (AL) amyloidosis is a malignant amyloidoses involving the proliferation of bone marrow plasma cell clones and the secretion of unstable immunoglobulin-free light chains (FLC) that aggregate into amyloid fibrils and infiltrate peripheral tissues causing end-organ damage. Disease presentation can be clinically non-specific, and early diagnosis and treatment initiation are critical, while cardiac involvement confers a worse prognosis.^1^ While AS is recognized to be associated with the transthyretin subtype of amyloidosis (ATTR), we present an interesting case of isolated aortic valve AL amyloidosis detected following surgical aortic valve replacement, illustrating how other amyloidosis subtypes can affect the aortic valve.

## Summary figure

**Figure:**
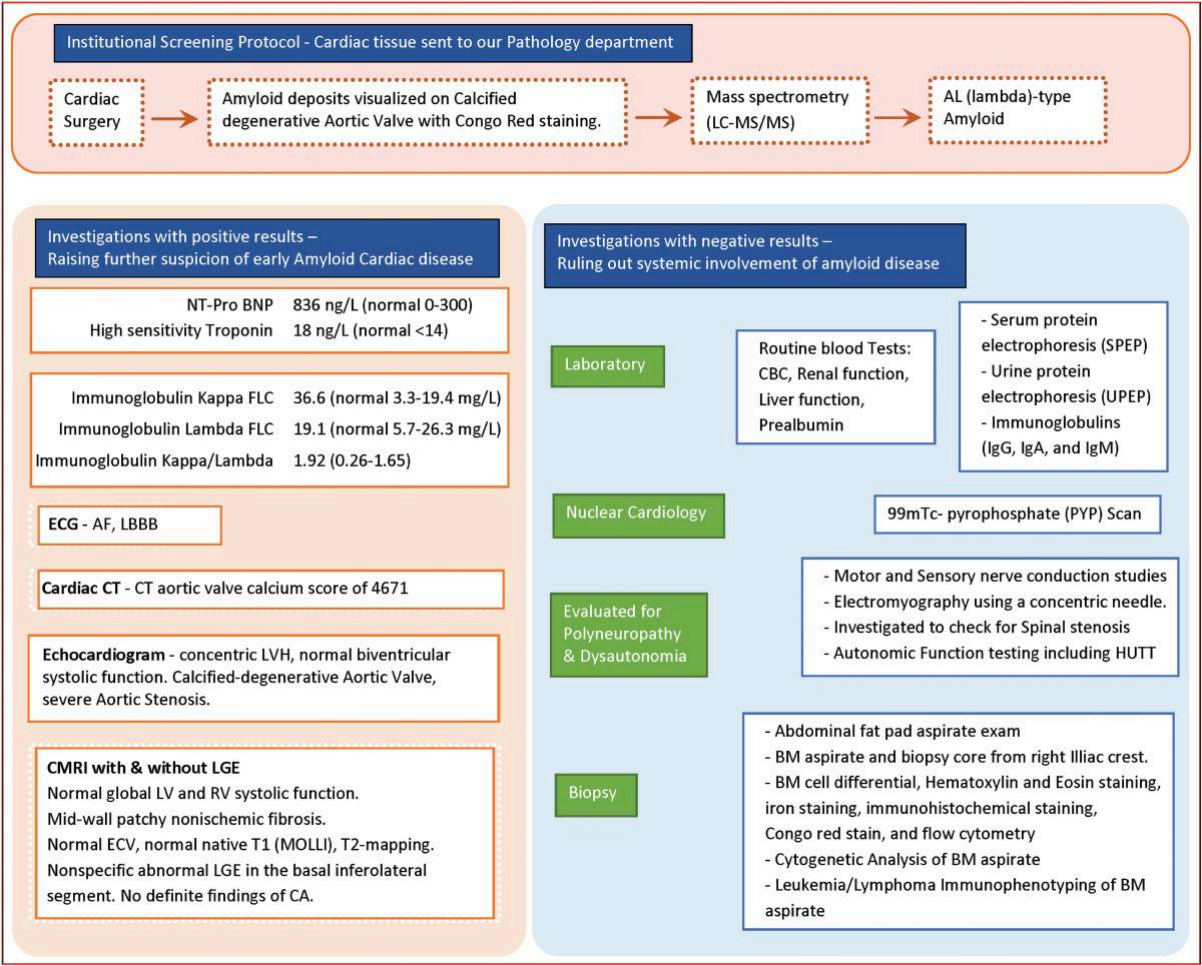


## Case report

**Figure 1:**
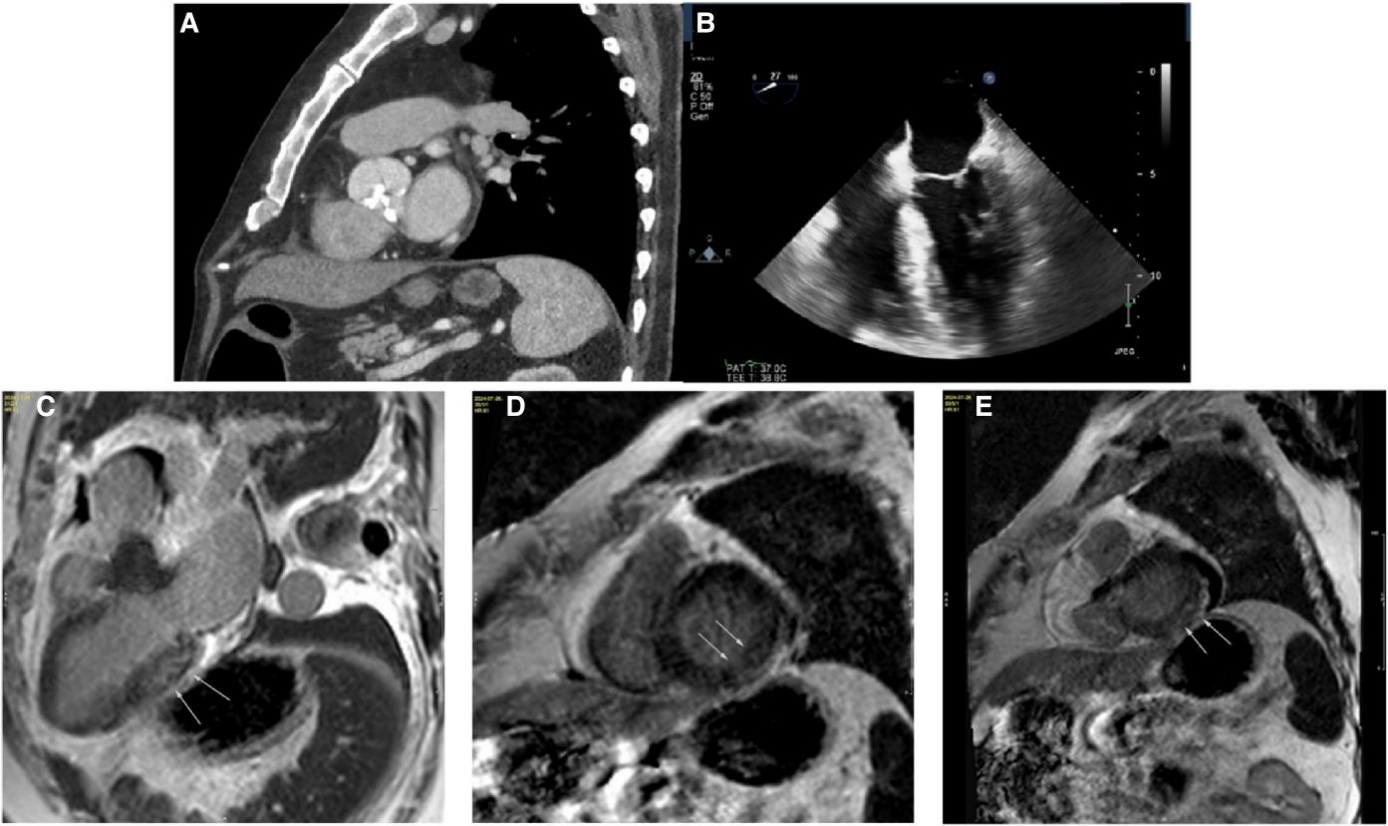
Cardiac imaging. Upper Panel: (*A*) Pre-operative chest computed tomography scan in the sagittal plane demonstrating a heavily calcified aortic valve (left). (*B*) transoesophageal echocardiography demonstrating increased concentric wall thickness with preserved biventricular systolic function (right). Lower Panel: Cardiac magnetic resonance imaging (*C*) Long axis 3 chamber view, white arrows showing patchy mid wall enhancement (non-ischemic fibrosis). (*D*) and (*E*) Short axis view, white arrows highlighting combination of patchy mid wall and subepicardial enhancement on the basal inferolateral segment of the left ventricle wall.

A 68-year-old Caucasian male with degenerative severe AS underwent uncomplicated surgical aortic valve replacement with implantation of a 25 mm Carpentier-Edwards Magna Ease bioprosthesis after presenting with a several-month history of worsening dyspnoea on exertion. This was his only significant symptom, with no other signs or symptoms of systemic illness. His pre-operative electrocardiogram demonstrated atrial fibrillation with a left bundle branch block QRS morphology. A transthoracic echocardiogram revealed a heavily calcified aortic valve, with significant calcification of the right coronary leaflet causing restricted motion and fusion between right and non-coronary cusps causing functionally partial bicuspid morphology. The mean systolic transvalvular Doppler gradient of 37 mmHg and peak gradient of 75 mmHg with a calculated aortic valve area of 0.83 cm^2^. There was increased concentric left ventricular wall thickness with normal biventricular systolic function (*Figure 1*). His medical history also included diabetes mellitus type 2, hyperlipidaemia, arterial hypertension, and Parkinson’s disease, all well controlled with medication.

**Figure 2:**
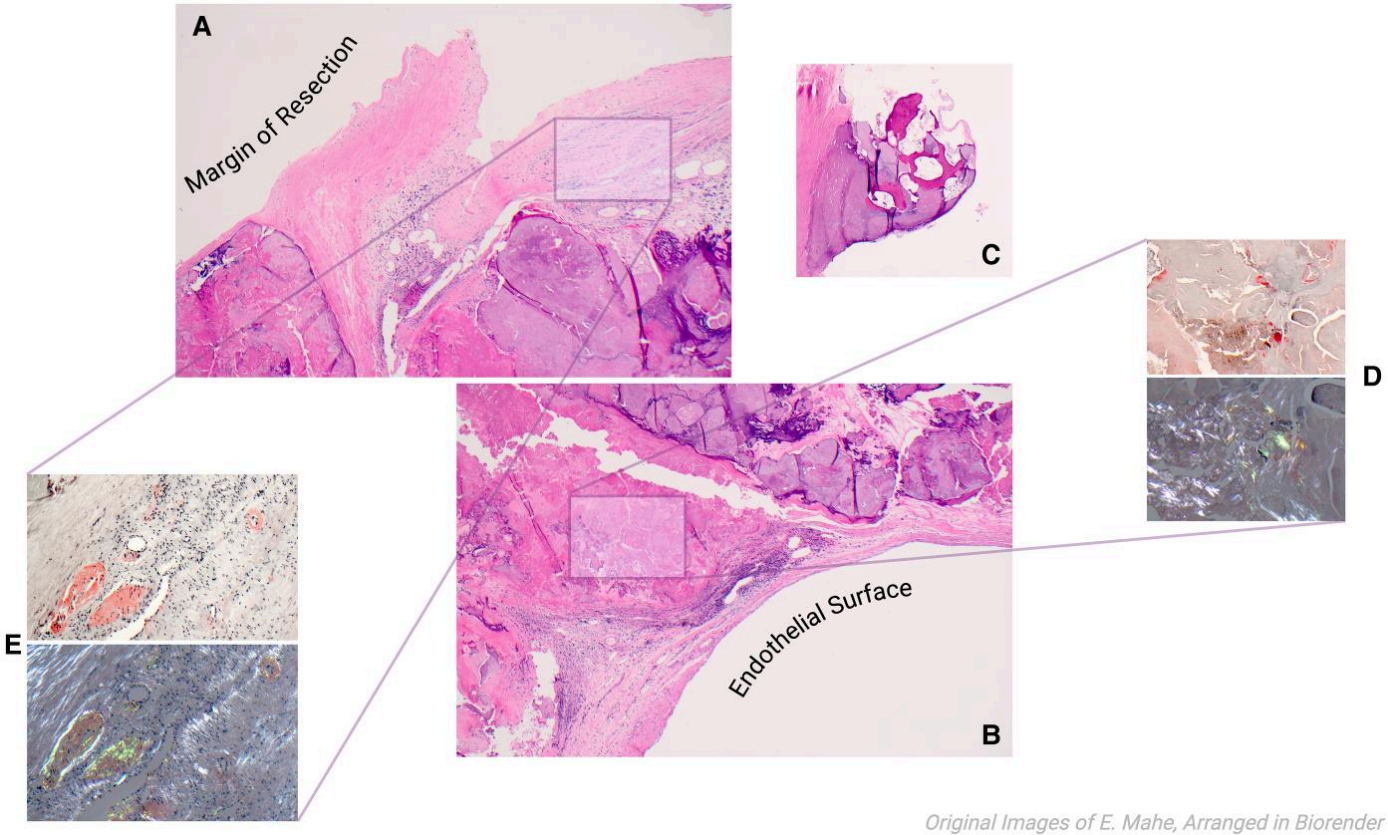
Pathology examination of the excised Aortic Valve. (*A*) and (*B*): Hematoxylin & Eosin (40×) photomicrographs, highlighting the width of the resected valve, sliced along its long axis, highlighting the endothelial surface and margin of resection. (*C*): Dystrophic ossification within the areas of degenerative change (Hematoxylin & Eosin; 40×). (*D*): Amyloid deposition, with morphological features typical of indeterminate amyloid deposition seen in association with degenerative change (Upper Panel: Congo red stain, unpolarized, 40×; Lower Panel: Congo red stain, polarized, 40×). (*E*): Perivascular and interstitial amyloid deposition, with morphological features typical of systemic amyloidosis (Upper Panel: Congo red stain, unpolarized, 100×; Lower Panel: Congo red stain, polarized, 100×).

During surgery, macroscopic inspection of the aortic valve confirmed heavy calcification. It was sent for pathology analysis as per standard clinical protocol. Microscopic examination demonstrated calcification, ossification, and fibrosis using ‘Musto-Movat’ staining, along with elastic fibres. Congo red staining performed to evaluate for amyloid deposits as per institutional practice was positive (*Figure 2*). Cardiac magnetic resonance imaging (CMR) was then performed and was negative for myocardial amyloid infiltration. Nuclear scintigraphy with technetium ^99m^Tc-pyrophosphate (PYP) was also negative for transthyretin uptake. Protein subtyping of the aortic valve tissue by mass-spectrometry revealed lambda AL-amyloidosis. An extensive work-up (*Summary figure*) followed to identify other organs potentially affected, including bone marrow and fat pad biopsy, and all tests came back negative. No monoclonal protein was identified by serum and urine analysis.

Monoclonal protein tests were repeated 8 weeks later and were unchanged. After a multidisciplinary discussion, cardiac biopsy was not performed, and it was decided not to offer further treatment for AL amyloidosis due to the absence of systemic involvement or other symptoms. At 9 months follow-up the patient was doing well and denied any symptoms. At 12-month clinic follow-up patient remains asymptomatic, repeat laboratory testing and CMRI with and without Gadolinium enhancement did not show any significant interval change compared with the initial study. He will continue to be closely monitored by a haematologist for evidence of disease progression with clinical and serum/urine surveillance, and a repeat CMR in 1-years’ time in case the need for treatment arises in future.

## Discussion

AS and CA may coexist, creating a diagnostic challenge as the clinical presentation for these conditions may overlap. The estimated prevalence of CA among patients presenting with AS is 9% to 16%,^2^ with the vast majority having transthyretin subtype (ATTR). This is particularly well reported among patients referred for transcatheter aortic valve replacement in particular, given the older age of this population and the association with the age-related wild-type (ATTRwt) subtype.^3^ A retrospective analysis of a prospective registry comprising 976 patients showed the crude prevalence of AS among patients with CA was 26% in ATTRwt, 8% in inherited variant (ATTRv), and 5% in AL,^4^ while moderate to severe AS was seen in 19%, 5% and 3% of patients with ATTRwt, ATTRv, and AL subtypes respectively.

Screening for CA in patients with AS is often not performed routinely in the absence of recognized amyloidosis ‘red flags’^5^ (such as extracardiac, electrocardiographic, cardiac biomarker or echocardiography or CMR findings). Elevated myocardial T1 with diffuse hyperenhancement in CMR represents a direct marker of cardiac amyloidosis infiltration is a strong predictor of mortality.^6^ It correlates well with systolic and diastolic dysfunction and is considered highly sensitive for detecting ‘early’ disease. Interestingly, our patient’s native T1-mapping and post-contrast extracellular volume quantification were normal with non-specific late gadolinium enhancement visualized in the basal inferolateral segment. In the absence of more definitive evidence of myocardial involvement or systemic amyloidosis involvement, or any notable symptoms after surgery, it was decided through multidisciplinary team discussion not to proceed with cardiac biopsy, and not to offer disease-modifying treatment such as chemotherapy for AL-amyloidosis, and to adopt a monitoring and surveillance approach to management. This will include serum and urine screening for the presence of monoclonal protein in addition to annual cardiac imaging with either echocardiogram or CMR, in addition to clinical follow-up and more routine investigations such as electrocardiogram and cardiac biomarkers. Any abnormalities detected that would suggest progression to systemic AL amyloidosis would prompt further invasive work-up.

To our knowledge, this is the first case report in the literature, describing isolated aortic valve AL-amyloidosis deposition. This case demonstrates that amyloidosis deposits may be present in stenotic aortic valves even when pre-operative clinical suspicion for cardiac amyloidosis is low. In light of this, our centre’s pathology laboratory has adopted a low threshold for screening for amyloid deposition using Congo red and/or sulfated-alcian blue stains. To facilitate histopathology assessment, aortic valve specimens are serially sectioned perpendicular to the valve surface. These are fixed in 10% neutral-buffered formalin, processed and embedded on edge in accordance with standard histopathological practice. Hematoxylin & eosin (H&E) stain evaluation facilitates initial histomorphological assessment. A Musto-Movat pentachrome stain facilitates the assessment of underlying elastic and collagen structures. Amyloid deposits identified by staining that are outside of areas of degenerative change are routinely evaluated by mass spectrometry to facilitate amyloid subtyping (performed by Mayo Clinic Laboratories). Based on historical mass spectrometry subtyping results, our laboratory screening approach suggests a false-positive rate of 2.7% Importantly, this approach for regular screening of aortic valve tissue for amyloid deposits is not presently endorsed in consensus statements or guidelines at the national or international level, and has been adopted by our institution based upon clinical experience regarding the frequency of amyloidosis deposits identified. This approach requires further research and validation before more widespread adoption can be considered.

While CA in AS is recognized to occur predominantly with the ATTR type, this case further elucidates the complex relationship between AS and amyloidosis, and importantly, that other subtypes in addition to ATTR can be involved. This case also highlights the importance of a multidisciplinary team approach to management and clinical decision-making in amyloidosis care, in particular for complex or unusual presentations such as this. This case relied on specialist input from cardiology, haematology, imaging and laboratory and pathology medicine to come to a consensus management plan, and ongoing collaboration will be required in the ongoing monitoring and follow-up of this patient.

## Data Availability

Data used in this paper is available to readers upon request.

## Lead author biography

Dr Rajin Choudhury is a Cardiologist with a special interest in cardiac electrophysiology, rare cardiomyopathies, and cardiac Imaging. He is at present in a clinical fellowship in Cardiac Amyloidosis at the Libin Cardiovascular Institute (Alberta Health Service), Cumming School of Medicine, University of Calgary, Calgary, Alberta, Canada.

**Consent:** The authors confirm that a written consent for submission and publication of this case report including images and text was obtained from the patient in compliance with COPE guidance.

**Funding:** None declared.
